# The relationship between the plasma proinflammatory cytokine levels of depressed/anxious children and their parents

**DOI:** 10.1038/s41598-021-90971-4

**Published:** 2021-06-03

**Authors:** Tomer Mevorach, Michal Taler, Shira Dar, Maya Lebow, Irit Schorr Sapir, Ron Rotkopf, Alan Apter, Silvana Fennig, Alon Chen, Abraham Weizman, Maya Amitai

**Affiliations:** 1 Department of Psychological Medicine, Schneider Children’s Medical Center of Israel, 14 Kaplan Street, 4920235 Petach Tikva, Israel; 2 The Pediatric Molecular Psychiatry Laboratory, Sheba, Tel Hashomer, Ramat Gan, Israel; 3 The Ruhman Family Laboratory for Research on the Neurobiology of Stress, Department of Neurobiology, Weizmann Institute of Science, Rehovot, Israel; 4 Department of Stress Neurobiology and Neurogenetics, Max-Planck Institute of Psychiatry, Munich, Germany; 5 School of Psychological Sciences, Tel Aviv University, Tel Aviv, Israel; 6 Bioinformatics Unit, Life Sciences Core Facilities, Weizmann Institute of Science, Rehovot, Israel; 7 Sackler Faculty of Medicine, Tel Aviv University, Tel Aviv, Israel; 8 Laboratory of Biological Psychiatry, Felsenstein Medical Research Center, Petach Tikva, Israel; 9 Research Unit, Geha Mental Health Center, Petach Tikva, Israel

**Keywords:** Cytokines, Psychology

## Abstract

Recent studies suggest immune function dysregulation in depression and anxiety disorders. Elevated pro-inflammatory cytokines may be a marker for immune system dysregulation. No study assessed the correlation between the levels of cytokines in children and adolescents with depression/anxiety disorders and their parents. In this study, 92 children and adolescents (mean age 13.90 ± 2.41 years) with depression and/or anxiety disorders were treated with fluoxetine. Blood samples were collected before initiation of treatment. One hundred and sixty-four of their parents (mean age 50.6 ± 6.2 years) and 25 parents of healthy children (mean age 38.5 ± 6.2 years) also gave blood samples. Plasma levels of three pro-inflammatory cytokine (TNF-α, IL-6, IL-1β) were measured by enzyme linked immunosorbent assays (ELISA) and compared between depressed/anxious children and their parents. We also compared cytokine levels between parents of children with depression/anxiety and control parents. Mothers of depressed children had higher TNF-α levels than mothers of controls. No significant difference was detected in the fathers. A positive correlation was found between the IL-1β levels of the depressed/anxious boys and their mothers. No such correlation was observed in the fathers. Our conclusions are that higher levels of proinflammatory cytokines may indicate immune system activation in mothers in response to the distress associated with having depressed/anxious offspring. The correlation between IL-1β levels in the mothers and their depressed/anxious children may indicate familial vulnerability to depression and anxiety. Our observation highlights the need for a better understanding of sexual dimorphism in inflammatory responses to stress.

## Introduction

Mood disorders are very common psychiatric disorders in children and adolescents. Recent studies have suggested that immune function may be dysregulated in children with depressive and anxiety disorders^1^. Cytokines are key players in numerous inflammatory processes. Upon recognizing an intrinsic or extrinsic danger signal, innate immune cells start to produce a variety of cytokines. The synthesis and regulation of serum levels of cytokines has been extensively studied, showing the significance of both environmental and genetic influences in this process^2^.

Anxiety and Depression have a considerable genetic component^3,4^. A heritable component in the variation of cytokine production would indicate that simultaneous occurrence of these conditions, (i.e. depression and anxiety), in parents and offspring might be caused by a heritable inflammatory characteristic. Thus, it may be postulated that inflammation may contribute to the co-occurrence of these conditions in parents and their offspring.

Few studies addressed the issue of the heritable component of inflammatory processes: de Craen et al. applied an extended twin study approach to assess heritability estimates of interleukin (IL)-1β, IL-1ra, IL-10, IL-6, and TNF-α production capacity^5^. Others assessed the heritable component in several inflammatory conditions such as asthma^6^. To our knowledge, there is only one in vitro study that evaluated the levels of cytokines in parents of children with depression, in an attempt to elucidate the genetic vs. environmental factors implicated in the dysregulation of the immune system in depression^7^. In this study, parents who were higher in empathy showed greater inflammatory cytokine production if their children also reported high levels of depressive symptoms, but lower cytokine production if their children reported low levels of symptoms.

The present study intends to dissect the familial versus the environmental influences on pro-inflammatory plasma cytokines. Three key cytokines involved in the human inflammatory response were assessed in children with depression and/or anxiety disorders severe enough to warrant fluoxetine treatment and their parents As a control group, we recruited a group of parents of healthy children. Our aims were to see whether: (1) differences exist in cytokine levels between parents of children with depression and/or anxiety disorders (parents of patients, PPs) vs. parents of healthy children (parents of controls, PCs); (2) cytokine levels in children and adolescents with depression/anxiety disorders are positively correlated with cytokine levels in their parents; (3) cytokine levels in parents of depressed/anxious children (PPs) are correlated with severity of psychopathology of their children. Our first hypothesis was that higher pro-inflammatory cytokines will be observed in the PPs vs. the PCs. This hypothesis assumes that PPs are experiencing chronic stress due to their child’s condition, as has been shown in other studies examining parental stress associated with a serious mental illness^8^. This chronic stress might cause elevation in pro-inflammatory cytokines^9^. Alternatively, a familial predisposition to anxiety/depression, mediated through inflammatory pathways, might cause higher cytokines in PPs vs. PCs. Our second hypothesis was that a positive correlation will be found between PPs’ and their children’s’ pro-inflammatory cytokine levels. This assumption is based on the notion that these children have a familial predisposition to immune system dysregulation in response to environmental challenges (such as stress). It is possible that there is an association between the parents and their offspring regarding immune system dysregulation which may play a role in the susceptibility to depression/anxiety. Alternatively, a correlation between PPs and their children’s cytokines may be related to the parents’ emotional response to their child’s stress. It has been previously shown that parents of depressed children, and especially mothers, reported higher parenting stress^10^. Our third hypothesis was that more severe psychopathology in the children will be accompanied with higher cytokine levels in the parents. We speculated that higher cytokine levels in the children reflect more severe psychopathology^11^, and this psychopathology causes more stress in the parents, eventually leading to higher levels of cytokines also in the parents. An alternative speculation is that higher cytokine levels in the parents, as a marker of immune system dysregulation, is genetically passed on to their children and causes immune system dysregulation and in turn more severe psychopathology in their children.

## Methods

### Study design

We approached patients with consecutive admissions to the psychiatric outpatient department at a university-affiliated pediatric medical center. Children and adolescents with depression and/or anxiety disorders severe enough to warrant pharmacological monotherapy with fluoxetine gave blood samples before initiation of fluoxetine treatment. Biological parents of these children and adolescents were recruited as the study group. All the biological parents were assessed for medical illnesses and medications. Parents with known immune, endocrine, or metabolic conditions such as autoimmune disorders, Inflammatory Bowel Disease (IBD), severe infection or HIV-AIDS were excluded from participation in the study. Also, treatment with immunomodulatory medications such as steroids, interferon or asthma related inhalant steroids and non-steroidal anti-inflammatory drugs (NSAIDs) was a cause for exclusion from participation in the study.

Parents of healthy children from the hospital staff were recruited as a control group. All of the control group volunteers were parents to healthy children and adolescents aged 6–18 years that did not have any major psychiatric diagnosis including depression or anxiety (as assessed by a child psychiatrist).

### Evaluation

The depressed/anxious children were assessed after 8 weeks of treatment with fluoxetine. Response to treatment was monitored with the Clinical Global Impressions-Improvement (CGI-I) scale^12^, Children’s Depression Rating Scale-Revised (CDRS-R)^13^, the Beck Depression Inventory (BDI)^14^, and the Screen for Child Anxiety-Related Emotional Disorders (SCARED)^15,16^.

At the end of 8 weeks of treatment, a child was considered a responder if CGI-I = 1 or 2, or a non-responder if CGI-I ≥ 3.

The parents of both groups (the depressed/anxious children and the healthy children) filled out questionnaires regarding demographic details. Psychopathology of the parents was assessed using the Family History Screen (FHS)^17^.

As it has been previously shown that Socioeconomic status (SES) affects inflammatory pathways^18^, we determined SES level of the PPs and the PCs. A SES grade was assigned according to profession and was divided to 3 levels: high (administrative), intermediate (professional or executive), and low (clerical or support) grades. This measure is a comprehensive marker of SES circumstances and is related to salary, social status, level of responsibility at work, and future pension^19^. Ethnic background was determined according to the country of origin of the grandfathers and grandmothers and was divided into 5 regions (European, African, Asian, Americas and Oceania and mixed) as was suggested for Israeli epidemiological studies^20^.

### Procedure

The study was approved by the Helsinki committee of Schneider Children Medical Center of Israel (SCMCI), and informed consent was obtained from the treated subjects and their parents. After a confirmatory diagnostic assessment, all subjects were treated with fluoxetine. The control parents also signed an informed consent. All experiments in this study were performed in accordance with the relevant guidelines and regulations.

### Subjects

Ninety-two children, 35 (38%) boys and 57 (62%) girls, aged 13.90 ± 2.41 years, were recruited to participate in this study. The children were diagnosed either with depression alone (8%), anxiety disorder alone (20%) or both (72.8%). One hundred and sixty-four of their biological parents were recruited. Out of these, five parents were excluded due to chronic illnesses (IBD, Multiple Sclerosis, Breast Cancer), leaving the sample with 159 parents: 79 mothers (mean age 43.67 ± 4.82 years) and 80 fathers (mean age 46.92 ± 6.83 years). Within the PPs, 10 father (12.5%) and 29 mothers (37%) had a lifetime history of depression; 13 (16%) fathers and 16 (20%) mothers had a lifetime history of anxiety disorder.

Twenty-five volunteers were recruited to the PCs group: 12 mothers (mean age 46.42 ± 3.58 years) and 13 fathers (mean age 35.69 ± 6.21 years). None of the parents in the control group reported chronic medical condition, or had a life-time history of anxiety and/or depression.

### Plasma cytokine assessment

Blood samples were collected from all parents and their depressed/anxious children at the baseline assessment of the children (i.e. before initiation of fluoxetine treatment). Blood samples were also taken from the parents of the healthy children. Blood samples were centrifuged immediately (1200 × rpm) at 4 °C for 10 min to obtain plasma. Plasma samples were separated into aliquots and stored at − 80 °C.

Duplicate samples were analyzed to determine levels of human cytokines TNFα, IL-6 and IL-1β in the same run, to avoid inter-assay variability. Cytokines were assayed on fifteen 96-well plates with samples from all groups distributed evenly. All cytokines were assessed with a sandwich enzyme linked immunosorbent assay (ELISA), based on a monoclonal-monoclonal antibody pair and a biotin-streptavidin amplification system (Siemens Medical Solutions Diagnostics, Los Angeles, CA), following the manufacturer's protocol. The laboratory staff was blinded to the clinical data and the clinical team was blinded to the laboratory data.

### Statistics

The Statistical Package for the Social Sciences (SPSS), version 26 for Windows (IBM corp, Armonk, NY) was used to create a database and conduct the statistical analyses. All three cytokines were not normally distributed (Kolmogorov–Smirnov test: TNFα: statistic = 0.095, df = 178, p value < 0.001; IL-6: statistic = 0.147, df = 178, p value < 0.001; IL-1β: statistic = 0.122, df = 178, p value < 0.001). Thus, data for all three cytokines were transformed into normal distribution using natural logarithms. To avoid batch-to-batch variation, all cytokine levels per batch were scaled to z-scores. Samples with cytokine levels below detection limit were assigned a value corresponding to the lowest detectable value in the assay (TNF-α: limit of detection [LOD] = 0.215; IL-6: LOD = 0.3384, IL-1β: LOD = 0.1157). Two-way ANOVA, Mann–Whitney and chi-square tests were used as appropriate; associations between the cytokine’s levels of the parents and clinical variables of the children, as well as correlation between cytokines values of the children group and the parents’ group were analyzed using Spearman's correlation or Pearson correlation, as appropriate. All analyses were two tailed and results expressed as Mean ± SD, with the level of significance set at 5%. p values were corrected for multiple testing using false discovery rate (FDR) where applicable.

## Results

### Cytokine measurements in patients’ parents (PPs) vs. the parents of controls (PCs)

Five hundred and seventy samples of PPs and PCs were analyzed in total (TNF-α: N = 188; IL-6: N = 193, IL-1β: N = 189). Ten samples had cytokine levels below detection limit and were assigned a value corresponding to the lowest detectable value in the assay (TNF-α: 4% of samples; IL-6: 0.5% of samples, IL-1β: 0% of samples).

No significant difference was observed between PPs and PCs in SES (Chi-square: χ^2^ = 4.24, df = 2, p value = NS). A signicifant difference in ethnic background was detected in the two groups (Chi-square: χ^2^ = 13.107, df = 4, p value = 0.023).

**Figure 1:**
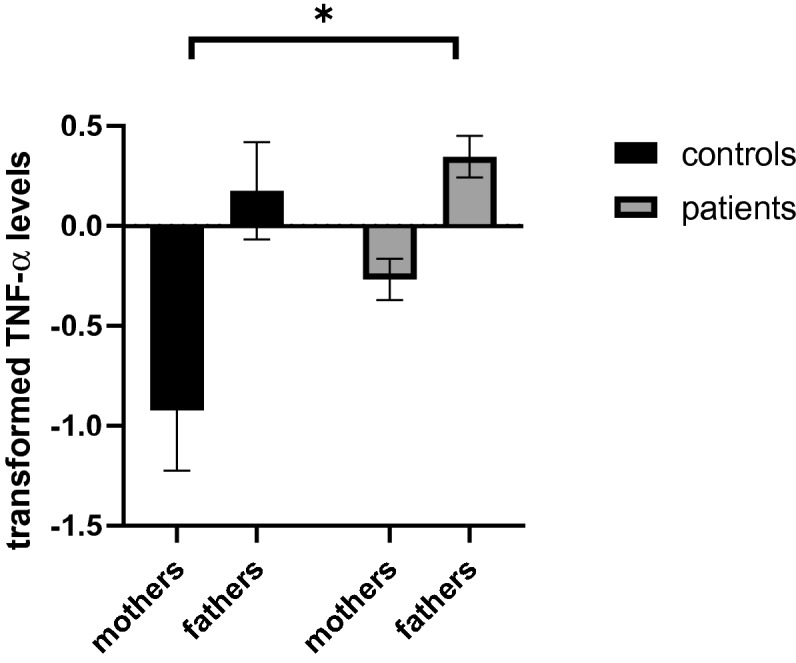
Transformed TNF-α levels in males vs. females. Levels were significantly lower in females than in males in both patients’ parents and control parents (male vs. females: 0.91 ± 0.44 vs 0.70 ± 0.44, F(1, 180) = 24.6, p < 0.001).

Transformed TNF-α levels were significantly higher in males than in females in both the PPs and the PCs (male vs. females: 0.91 ± 0.44 vs 0.70 ± 0.44, two-way ANOVA: F(1, 180) = 24.6, p value < 0.001; see Fig. 1).

No association was found between cytokine levels and age (data not shown). Thus age was not considered as a covariate.

No difference was found between the PPs and PCs in the three cytokines measured.

**Figure 2:**
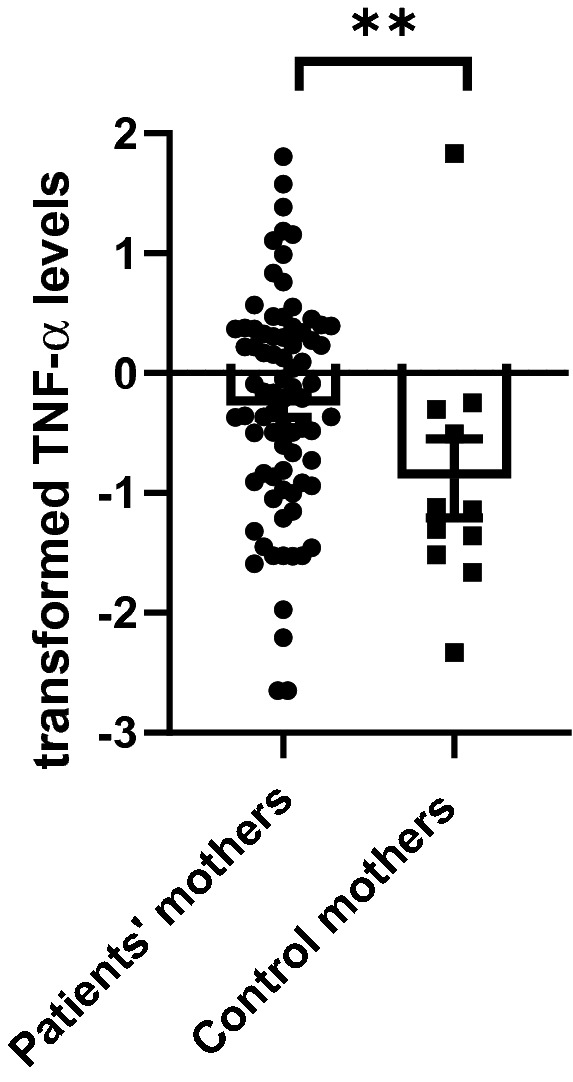
Transformed TNF-α levels in the patients’ group vs. the control group. Higher transformed TNF-α levels were found in the patients’ mothers vs. the control mothers (Mann–Whitney: p = 0.009, adj. p value = 0.027).

Higher transformed TNF-α levels were found in the PP mothers vs. the PC mothers (PPs vs. PCs mothers: − 0.267 + 0.918 vs. − 0.922 vs. 1.050, Mann–Whitney: p value = 0.009, adj. p value = 0.027, see Fig. 2). No difference was observed regarding IL-6 and IL1-β levels. No difference was observed between the fathers in all three cytokines measured (p value = NS for all). See Table 1 for the cytokine levels in PPs group vs. the PCs group.

**Table 1:** Transformed cytokine levels in the parents of the patients’ group vs. the parents of the control group.

	Patients parents (PPs), N = 159	Control parents (PCs), N = 25	Mann–Whitney
Transformed TNF-α levels: mothers	− 0.267 ± 0.918	− 0.922 ± 1.050	p value = 0.009 adj. p value = 0.027
Transformed TNF-α levels: fathers	0.347 ± 0.929	0.176 ± 0.877	p value = NS adj. p value = NS
Transformed IL-6 levels: mothers	0.065 ± 0.996	− 0.764 ± 1.425	p value = 0.056 adj. p value = NS
Transformed IL-6 levels: fathers	0.063 ± 0.882	0.013 ± 0.724	p value = NS adj. p value = NS
Transformed IL-1β levels: mothers	0.013 ± 0.942	0.334 ± 1.007	p value = NS adj. p value = NS
Transformed IL-1β levels: fathers	− 0.005 ± − 1.059	− 0.220 ± 0.690	p value = NS adj. p value = NS

### Cytokine measurements within the parents of the patients (PPs)

No differences were observed in cytokine levels between parents with depression and/or anxiety disorders vs. healthy parents (p value = NS for all, data not shown).

**Figure 3:**
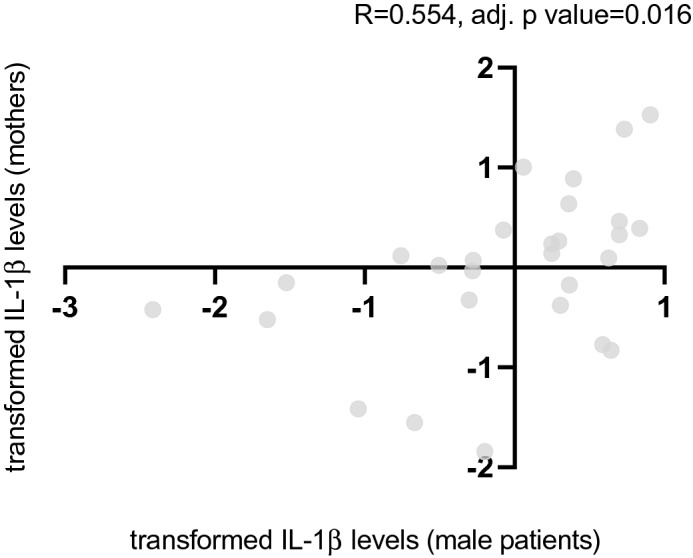
Correlation in transformed IL-1β levels between mothers and their depressed/anxious sons. A positive correlation was found between IL-1β levels in male patients and their mothers (Spearman correlation: R = 0.554, p value = 0.002, adj. p value = 0.016).

**Figure 4:**
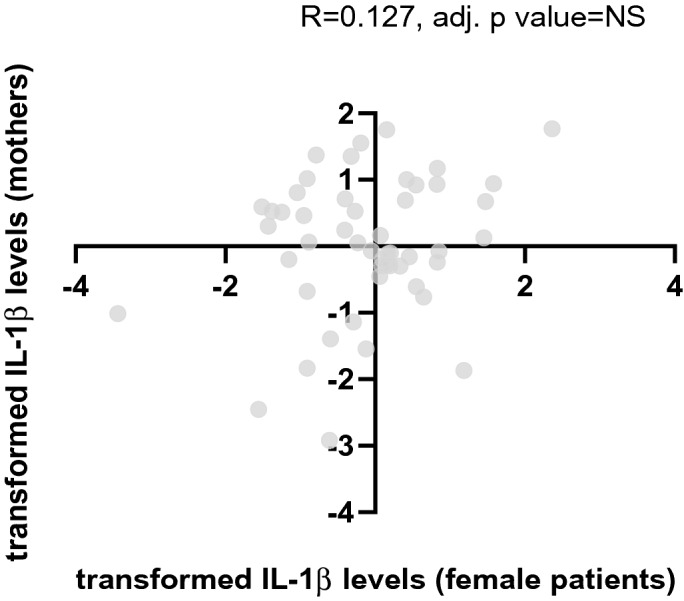
Correlation in transformed IL-1β levels between mothers and their depressed/anxious daughters. No correlation was found between IL-1β levels in female patients and their mothers (Spearman correlation: R = 0.127, p value = 0.38, adj. p value = NS).

No correlation was found in plasma cytokine levels between patients and their parents (Spearman correlation, p value = NS for all). When dividing the patients to males and females, a positive correlation was found between IL-1β levels in male patients and their mothers (Spearman correlation: R = 0.554, p value = 0.002, adj. p value = 0.016, see Fig. 3). No such correlation was observed between mothers and female patients (Spearman correlation: R = 0.127, p value = 0.38, adj. p value = NS, see Fig. 4).

A positive correlation was found between mothers’ TNF-α levels and CDRS-R scores in their sons but not with those in their daughters. However, the significance of the correlation between the mothers and their sons was lost following FDR correction (Spearman’s correlation: R = 0.414, p value = 0.028, adj. p value = NS). No correlation was found between fathers’ TNF-α levels and male or female patients with regard to CDRS-R scores.

A positive correlation was found between father’s TNF-α levels and SCARED score in female patients but not in their sons. The correlation between the fathers and their daughters lost significance following FDR correction (Spearman’s correlation: R = 0.286, p value = 0.042, adj. p value = NS). No correlation was found between mother’s TNF-α levels and males or female patients with regard to SCARED scores.

No difference was observed in the parents’ cytokine levels between parents of children with suicidality at baseline vs. children without.

The plasma levels of the cytokines did not differ between parents of responders vs. parents of non-responders, or parents of children with adverse events vs. parents of children without.

## Discussion

Although no differences were detected in the plasma pro-inflammatory cytokine levels between PPs and PCs, a significant difference was observed in TNF-α levels between mothers of PPs and PCs. Moreover, a positive correlation was observed in IL-1β between the mothers of PPs and their sons. In this study we examined the levels of three pro-inflammatory cytokines in parents of children with depression/anxiety disorders in order to dissect the role of genetic vs. environmental factors in the contribution of immune system dysregulation to anxiety and depression. There is accumulating evidence suggesting that immune dysfunction may contribute to the pathogenesis of depression^21^, and thus we expected differences in immune markers between parents of healthy children vs. parents of children with psychopathology. However, No differences between patients’ parents and controls were observed in the pro-inflammatory cytokine levels when analyzing the whole group pf parents.

Surprisingly, when analyzing males and females separately, we did observe significant female-male differences in our whole data set:higher TNF-α levels were observed in males vs. females, both in the PPs and the PCs (Fig. 1). Sexual dimorphism in cytokine production in response to stress has long been described in the literature^22^. Higher cytokine levels in males vs. females were reported in both male humans and animals^23^. Thus, our finding is consistent with the current literature regarding sexual dimorphism, at least regarding TNF-α production. According to this finding, we checked for gender differences in all further comparisons.

Even though no differences were observed between patients' parents and control parents in the cytokine levels, when analyzing fathers and mothers separately, a statistically significant difference was observed between mothers of depressed/anxious children vs. mothers of healthy children, namely higher TNF-α levels in PP mothers vs. PC mothers. No such difference was observed in fathers. A finding that goes along with the higher TNF-α in the PPs was the correlation between IL-1β in PPs mothers and their depressed/anxious sons. Interestingly, no such correlation was observed between the mothers and their daughters. The discrepancy between the full sample and gender-specific results should be interpreted with caution, as it is possible that these findings reflect a type 1 error, especially since it is lacking a-priori theoretical rationale. Similarly, is it possible that the lack of relationship with fathers rather than mothers is actually reflective of the fact that fewer fathers participated in the study than mothers, and thus we were potentially underpowered to detect effects in the fathers. Despite these limitations, these finding are interesting and deserve attention.

The explanation to these observations may be broadly divided into environmental vs. genetic factors. The first explanation may be related to the psychological stress of the parents in response to their child’ psychopathology. Due to the child’s condition, there may be an elevation in stress levels in the home or tension between parent and child or worrying on the part of the parent. It is well known that stress, and specifically psychological stress, dramatically activates inflammatory processes such as microglial activation, cytokine release, the number and proportion of circulating T and B cells, alterations in natural killer cell numbers and cytotoxicity, and impairment of functional responses such as mitogen-induced cell proliferation^24^. Although no differences were observed in cytokine levels between parents with anxiety and/or depression vs. healthy parents, it is still possible that higher cytokine levels indicate corresponding higher levels of stress in the parents without fulfilling the DSM-5 criteria for depression or anxiety. It has been previously shown that chronic stress is associated with immune system’s non capacity to respond to neuro-hormonal signals that terminate inflammation. Meta-analysis of studies regarding controlled, acute psychologic stressors in humans identified increases in circulating inflammatory factors^25^. Specifically, it was demonstrated that chronic stress attenuated the immune system’s response to anti-inflammatory signals in parents of cancer patients^26^. Despite the differences between the two conditions (cancer vs. depression/anxiety), this study is an example of a situation where stress levels of the parents ascribed to their child’s condition affect the immune system. Thus, our finding may indicate that the parents of children with depression and or/anxiety experience stress in response to their children’s’ distress, especially before treatment initiation.

It is also possible that a shared environmental factor influences cytokine levels in the mothers and their children. However, this explanation is less likely since we would have expected that the same dysregulation would be observed in the fathers as well.

An alternative explanation lies in genetic factors: it may be that the higher levels of TNF-α indicate immune system activation in response to stress, and this dysregulation may indicate genetics vulnerability to these disorders in the offspring. Studies have shown that innate cytokine production capacity is under strong genetic control, at least in adult patients^27,28^. Heritability estimates of the production capacity of various cytokines range from 53 to 86% in non-diseased population^5^, indicating that the production of cytokines is under tight genetic control. It appears that at an early age, the capacity of the infant’s immune cells to produce pro- and anti-inflammatory cytokines is more influenced by maternal cytokines than by the genetic variation tested, which seem to have more influence at later age. One study showed a close relationship of mother and child cytokine production, especially IL-10 and IFN-γ, that was prominent in early life, before 1 year of age. This immunological relationship appeared to be independent of cytokine gene polymorphisms, suggesting that infant’s cytokine responses were more influenced by the environment shared with the mother during intra uterine and breastfeeding period^29^. However, as the child grows the genetic influence become more pronounced and it is possible that at the age range we are addressing the genetic component grows to be more prominent, at least with regard to the maternal immune system.

It is known that anxiety often aggregates within families, as children of parents with clinical anxiety are at an increased risk of developing anxiety-spectrum disorders throughout their lifespan^3^. Depression is also highly genetic^4^. This observed association might have an immunogenetic explanation. It is possible that the genetic susceptibility to anxiety/depression results from immune system dysregulation, present also in the mothers. Thus, it is possible that a heritable component in the variation of cytokine production would indicate the simultaneous occurrence of conditions, such as depression and anxiety.

Notably, only one cytokine was elevated in the mothers’ PPs vs. the PCs (i.e. TNF-a levels). TNF-α is a pro-inflammatory cytokine, regulating the acute phase response. It seems that TNF-α is a more sensitive marker for immune system over-activity than the two other pro-inflammatory cytokines, at least regarding the mothers’ distress. Interestingly according to one study, genetic influences (heritability) increase for TNF-α with age due to a decrease in unique environmental factors, meaning that genetic factors become more important in regulation of TNF-α during lifetime as compared to other cytokines^2^. Also, the correlation between the mothers and their sons was detected only regarding one cytokine (IL-1β). Interestingly, according to one study, IL-1β synthesis had the highest estimate heritability of 86% as compared to other cytokines^5^, and thus it may be that indeed this correlation points to a genetic susceptibility. However, it is unclear why this observation appeared only in the mothers and sons (and not in the daughters or the fathers).

Sexual dimorphism is observed in humans and animals from birth to death with regard to inflammation. Numerous studies have reported higher cytokine levels in both male humans and animals than in females. Estrogens and androgens are known to modulate inflammation, and their different levels in males and females may explain some of the sexual dimorphism observed^22,30,31^. This goes along with our finding of higher TNF-α levels in males compared to females.

The sexual dimorphism finding may be explained by two alternative explanations: the environmental vs. the genetic. The male–female differences may be caused be environmental factors: There may be a difference between mothers and fathers in the psychological reaction to the stress of their depressed/anxious children. Several studies have shown that mothers react with more stress to the psychopathology of children^32^, and as such are more prone to develop proinflammatory elevation in response to distress. Previously it has been shown that there is a distinct inflammatory response to social stress in females that may underlie the increased sensitivity that women demonstrate to social stressors and may promote the increased risk of stress-related psychiatric disorders^33,34^.This finding has also been recapitulated in animal models^22^. For instance, a recent study evaluated sex differences in the inflammatory response to an LPS injection 24 h after inescapable shock. Peripheral cytokine release was potentiated in females with a history of stress compared with stressed males or unstressed males or females^35^.

One of the possible mechanisms to the sex-specific inflammatory response to stress lies in the experimental evidence supporting a role for sex differences in stress-induced microglial activation. Microglial cells are capable of changing morphologically between a proinflammatory and anti-inflammatory state^36^. Microglia, when activated by stress, shift morphology states, leading to the secretion of proinflammatory cytokines such as Il-1β, IL-6, and TNF-α to neighboring cells^37^. Several studies have shown that acute or chronic restraint stress affected microglial morphology in a sex dependent manner. In the orbitofrontal cortex, basolateral amygdala, and dorsal hippocampus, chronic stress reduced microglial activation (as evidenced by morphology changes) yet upregulated a greater number of immune factors in females compared with males^38^. These findings provide evidence of sex differences in microglial morphology that may bias females toward greater neuroinflammatory responses in the context of stress, leading eventually to depressed mood^22^. Interestingly, maternal immune activation (MIA) was recently proved to be an environmental risk factor for psychiatric and neurological disorders with neurodevelopmental etiologies. MIA-exposed offspring show dissociable behavioral, transcriptional, brain network, and immunological profiles even under conditions of genetic homogeneity^39^.

It may be that mothers react with more psychological distress to the distress of the child than fathers. It has already been shown that mothers respond with more distress to a depressed child, while such a phenomenon did not occur among the fathers^10^. It may be that higher stress the mothers experience also promotes in turn higher stress in the offspring. Since heritability estimates of anxiety are modest^40^, environmental factors such as parental behaviors, modeling, and broader family functioning likely influence anxiety’s intergenerational transmission^41^.

Alternatively, genetic factors may explain also the sexual dimorphism observed. It may be that the immune-genetic component which exposed the mothers and their offspring to develop anxiety/depressive disorders is more pronounced in the sons than in the daughters. Further studies should address this question.

Some limitations of our study need to be acknowledged. First, our study had a cross-sectional design, which makes it impossible to draw any conclusions about causality. Thus, we are unable to conclude whether the cytokine production capacity is more likely to be a causal factor than the consequence of the stress experienced by the mothers. Another limitation is that we have no psychological measures of the parents, and thus we do not have objective measure for their levels of stress. Another limitation is that parents’ samples were collected only at baseline. Blood sample collection after the fluoxetine treatment in the children could indicate whether the cytokine levels of the PPs correlate with the degree of improvement of their children. Also, we have no cytokine measures of the healthy children (the children of the PCs), so we could not tell whether the results we found in the PPs are unique to this group. Another limitation in this study is the fact that parents of the children with depression and/or anxiety and the parents of healthy children are not age matched. Moreover, even though no difference was observed in SES between the groups, they differed in their ethnic background. However, it is unlikely that this difference affected our results. Aditionally, we have not assessed whether the patients were fasting or had eaten before blood sampling, which might affect cytokine levels. However, the strength of our study lies in the relatively large cohort and the unique design.

## Conclusions

Our findings may indicate immune system dysregulation which may predispose individuals to psychopathology. Alternatively, this may indicate higher stress levels in the mothers which may lead to immune dysregulation. Our findings indicate the need to evaluate the correlation of immune response to stress between parents and their offspring and highlight the need for a better understanding of sexual dimorphism in inflammatory responses in depression and anxiety disorders.
